# Primary clinical testing of the questionnaire “Brief questionary of quality of anxiety” in patients with generalized anxiety disorder

**DOI:** 10.1192/j.eurpsy.2022.986

**Published:** 2022-09-01

**Authors:** M. Morozova, G. Rupchev, D. Burminskiy, S. Potanin, T. Lepilkina, A. Beniashvili

**Affiliations:** 1 FSBI Research Center of Mental Health, Laboratory Of Psychopharmacology, Moscow, Russian Federation; 2 Moscow State University, Faculty Of Psychology, Moscow, Russian Federation

**Keywords:** generalized anxiety disorder, quality of anxiety, Questionnaire

## Abstract

**Introduction:**

The clinical differentiation of anxiety can play an important role, particularly in response to treatment. Patients with generalized anxiety disorders (GAD) reflect anxiety, therefore questionnaires are effective. Previous attempts to create a questionnaire assessing the quality of anxiety assessed only one aspect – tolerance to uncertainty (3). The new questionnaire covers such aspects of anxiety as behavioral manifestation, hypochondria, relation to cognition, personal trait and expectation from treatment.

**Objectives:**

Clinical testing of the questionnaire.

**Methods:**

38 GAD patients (total score of Hamilton depression rating scale 27±4.7), aged 42.5±13, 75% females and 38 healthy volunteers aged 36.5±11, 74% females. The questionnaire included 8 statements, (two of them have subparagraphs). The testing version does not include statement about expectations from treatment. It takes 10 minutes to fill it out.

**Results:**

The difference between groups were found in following statements:

“I am often told that a am worried about small things” (χ2 22 p=0.00001)–behavioral presentation of anxiety.

“When I am anxious, I find it difficult to concentrate” (χ23,6 p=0.059)–cognitive aspect.

“My anxiety is getting worse, when I can’t complete the task strictly according to the instruction” (χ2 13.6 p=0.0002) -obsessive aspect.

“My anxiety is getting worse, when something goes wrong” (χ2=9 p=0.002)-obsessive aspect.

“My anxiety is getting worse, when I need to make my own decision” (χ29 p=0.003)- narcissism

“My anxiety is getting worse when I have to hold back irritation or discontent” (χ24.2 p=0.04)-narcissism.

**Conclusions:**

Only part of statements differs GAD patients from healthy volunteers, but they cover different fields of mental functioning.

**Disclosure:**

No significant relationships.

